# Cyclic GMP-AMP Synthase Is Required for Cell Proliferation and Inflammatory Responses in Rheumatoid Arthritis Synoviocytes

**DOI:** 10.1155/2015/192329

**Published:** 2015-12-27

**Authors:** Yan Wang, Guo-Hua Su, Fang Zhang, Jing-Xue Chu, Yun-Shan Wang

**Affiliations:** ^1^The Medical Laboratory Diagnostics Center, Jinan Central Hospital Affiliated to Shandong University, Jinan, Shandong 250013, China; ^2^Department of Bone and Joint Surgery, Jinan Central Hospital Affiliated to Shandong University, Jinan, Shandong 250013, China

## Abstract

Rheumatoid arthritis (RA) is characterized by inflammatory cell infiltration, fibroblast-like synoviocytes (FLS) invasive proliferation, and joint destruction. Cyclic GMP-AMP synthase (cGAS) is a cytosolic DNA sensor that induces immune activation. In this study, we examined whether cGAS plays a role in RA FLS. In this study, cGAS was overexpressed in RA-FLS compared with OA FLS. TNF*α* stimulation induced cGAS expression in RA FLS. Overexpression of cGAS promoted the proliferation and knockdown of cGAS inhibited the proliferation of RA FLS. cGAS overexpression enhanced the production of proinflammatory cytokines and matrix metalloproteinases (MMPs) as well as AKT and ERK phosphorylation in TNF*α*-stimulated FLS. In contrast, cGAS silencing inhibited production of proinflammatory cytokines and matrix metalloproteinases (MMPs) as well as AKT and ERK phosphorylation in TNF*α*-stimulated FLS. These results suggest that cGAS activates the AKT and ERK pathways to promote the inflammatory response of RA FLS, and the development of strategies targeting cGAS may have therapeutic potential for human RA.

## 1. Introduction

Rheumatoid arthritis (RA) is a chronic systemic autoimmune disease characterized by proliferative synovitis, hyperplasia of the synovial tissues, and destruction of cartilage and bone. Fibroblast-like synoviocytes (FLS) are crucial in the development of arthritis [1, 2]. The activated FLS proliferation in RA joints and their secretions such as proinflammatory cytokines and matrix metalloproteinases (MMPs) play pivotal roles in the development of synovial hyperplasia, sustained inflammation, and joint destruction in arthritic joints [3].

Recently, several studies have recently revealed a role for type I interferons (IFNs) in the pathogenesis of RA patients [4]. Genetic variants associated with type I IFN pathway have been linked with RA development, as well as with clinical features [5, 6]. The cytosolic DNA sensor, cyclic GMP-AMP synthase (cGAS), has been reported to be required for activating IFN production via the STING pathway [7, 8]. Recent studies have implicated the key role of cGAS in autoimmune disease in the mouse model of Aicardi-Goutières syndrome and in the innate and adaptive immune responses in features of human lupus [9–11]. However, the role of cGAS in RA remains unknown. In this study, we explored whether cGAS is involved in the pathogenesis of human RA via affecting RA-FLS.

## 2. Material and Methods

### 2.1. Patients and Controls

Synovial tissues were obtained from 8 RA patients and 8 osteoarthritis (OA) patients who underwent knee arthroscopic or replacement surgery at our hospital. Serum samples were taken before the surgery from all patients. All the subjects fulfilled the 2010 American College of Rheumatology (ACR) criteria for the diagnosis of RA and OA [12, 13]. Informed consent was obtained from all patients and the study protocol was approved by the Ethics Committee of Our Hospital (IRB-S1126).

### 2.2. Isolation and Culture of FLS

Synovial tissues were minced into pieces of 2 to 3 mm in size and spread on the bottom of cell culture flasks in RPMI 1640 medium (Life Technologies, Carlsbad, CA, USA) at 37°C for 6 hours. Next, the tissues were incubated with complete RPMI 1640 medium supplemented with 10% fetal calf serum in a humidified atmosphere containing 5% CO_2_. The medium was changed every three to five days and nonadherent tissue pieces were carefully removed. FLS were grown further over four to six passages. To characterize the cytological phenotype of synovial cultures, the third passage cells were stained with mouse monoclonal antibodies (mAb) to human fluorescein isothiocyanate (FITC)-CD14 and phycoerythrin (PE)-CD90 (eBioscience, San Diego, CA, USA) and showed 2.8% CD14 and 97.0% CD90 expression, as measured by flow cytometry.

### 2.3. Quantitative RT-PCR (qRT-PCR)

Total RNA from FLS was extracted using TRIzol Reagents (Invitrogen Life Technologies, Carlsbad, CA, USA) according to the instructions. Complementary DNA was synthesized using a reverse transcription system (Takara, Dalian, China), and the cGAS transcript was detected by PCR amplification using cGAS-specific primers (forward: 5′-TAACCCTGGCTTTGGAATCAAAA-3′; reverse: 5′-TGGGTA CAAGGTAAAATGGCTTT-3′). *β*-actin was used as an internal reference gene control using specific primers (forward: 5′-ACGCATCTGGCAGTACGTCTA-3′; reverse: 5′-CCG GTTCTTACTCGGGTTGAG-3′). Real-time quantitative PCR was performed using 1 *μ*L of complementary DNA per well, TaqMan Master Mix (Applied Biosystems, Foster City, CA, USA), and results were evaluated using the ΔCT method and the calculated number of copies was normalized to that of *β*-actin copies in the same sample.

### 2.4. Western Blot

The collected cells were suspended in Laemmli sample buffers (Bio-Rad, Hercules, CA, USA) containing 5%  *β*-ME, followed by boiling for 5 min. The supernatants were collected as cell lysates, and the protein concentration was measured using BCA protein assay kit (Pierce, Appleton, WI, USA).

Equal amounts of protein samples (50 *μ*g) were loaded on a 10% SDS-PAGE and transferred onto nitrocellulose membranes. The membranes were blocked with 5% nonfat milk in TBST buffer for 2 h at room temperature and subsequently incubated with primary antibodies at 4°C overnight, respectively. It was followed by incubation with respective HRP-conjugated secondary antibodies, and the bands were developed with ECL substrate and exposed to X-ray film or scanned using Tannon 5200 (Tanon, Beijing, China) according to the manufacturer's instructions. Blots were analyzed for band intensities using ImageJ software. For quantification, the relative abundance of each protein was normalized to *β*-actin control. The following primary antibodies were used: anti-*β*-actin, anti-cGAS, anti-phospho-AKT, AKT, anti-phospho-ERK1/2, anti-ERK1/2 (all from Cell Signaling Technology, Danvers, MA, USA). Horseradish peroxidase-conjugated anti-mouse or anti-rabbit IgG (Santa Cruz Biotechnology, CA, USA) was used as a secondary antibody.

### 2.5. Construction of Lentivirus Vectors

TRC pLKO.1 lentiviral vectors targeting human cGAS (Thermo Fisher Scientific, Cat. # 129125) were used. Scrambled shRNA was cloned into the same vector and was used as control. Furthermore, full-length cGAS cDNA was cloned into lentiviral vectors pWPI and linked to IRES-GFP. The pWPI vectors expressing GFP only were used as the controls. The vectors were amplified in HEK293 cells and purified by CsCl gradient ultracentrifugation. Finally, acquired lentivirus particles were dialyzed at 4°C against sterile virus buffer and stored at −80°C.

### 2.6. Cell Proliferation Assay

Cell proliferation was determined as described by Zhang et al. [14] by using the CellTiter 96 Aqueous One Solution Cell Proliferation Assay kit (Promega, Beijing, China), according to the manufacturer's instructions. Briefly, cells were plated at 1 × 10^3^ cells/well in 96-well plates and cultured for different periods. At the end of each period, 20 *μ*L MTS was added to each well and then incubated at 37°C for 4 hours. Plates were read at 490 nm on a spectrophotometric plate reader (Bio-Rad, Hercules, CA, USA) with a reference wavelength at 650 nm. The index for stimulating cell proliferation was calculated as intervention group optical density (OD) value/blank control group OD value.

### 2.7. Quantification of Proinflammatory Cytokines and MMPs

Protein levels of human IL-1*β* and IL-6 in cell-free FLS supernatants were measured using commercially available enzyme-linked immunosorbent assay (ELISA) kits (NeoBioscience, Beijing, China) according to the manufacturer's instructions. MMP-1 was quantified by fluorescence assay using the Human Active MMP-1 Fluorokine E kit according to the manufacturer's protocol (R&D Systems, Minneapolis, MN, USA). MMP-3 expression was determined using a Human MMP-3 Quantikine ELISA kit (R&D Systems, Minneapolis, MN, USA).

### 2.8. AKT/ERK Activation ELISA Assay

The AKT/ERK Activation InstantOne ELISA kit (eBioscience, San Diego, CA, USA) was used for preliminary measurement of phosphorylated human ERK1/2 and AKT in RA FLS lysates according to the user manual. Briefly, RA FLS (2 × 10^5^ cells) were infected with viral vectors for 48 h and then treated with human TNF*α* (100 ng/mL) for an additional 30 min. The cells were lysed with the lysis buffer mix and the prepared sample lysate (50 *μ*L) was added to the strip-well ELISA assay plate. After adding the antibody cocktail (50 *μ*L), the plate was incubated for 1 h at room temperature on a microplate shaker. After washing, the wells were incubated with the detection reagent (100 *μ*L) for about 15–20 min, followed by stopping the reaction with the stop solution. Finally, the absorbance at 450 nm was measured.

### 2.9. Statistical Analysis

Data are expressed as the mean and SEM (standard error of the mean). Each result represents the mean of three independent experiments. Comparisons were conducted using Student's *t*-test (2 groups) or the ANOVA (more than 2 groups) for statistical analysis. All analyses were performed using SPSS 17.0 (IBM Corp, Armonk, NY, USA). *P* values less than 0.05 were considered significant.

## 3. Results

### 3.1. cGAS Was Overexpressed in RA-FLS Compared with OA FLS

**Figure 1:**
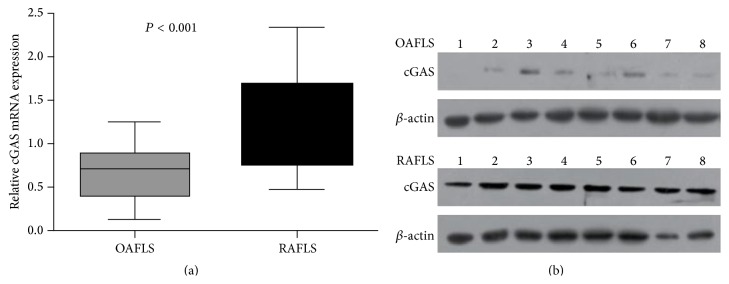
Expression of cGAS is enhanced in RA-FLS compared with OA FLS. (a) qRT-PCR analysis of cGAS expression in 8 RA-FLS and 8 OA FLS. Quantitative analysis of cGAS expression was normalized to *β*-actin expression. (b) Western blotting identified the expression of cGAS in RA-FLS and OA FLS.

The relative expression of cGAS mRNA in cell lines and tissues was quantified by qRT-PCR in FLS from eight RA and OA patients. When normalized to *β*-actin, the mean expression levels of cGAS mRNA were higher in RA FLS than in OA FLS (*P* < 0.001) (Figure 1(a)).

In order to further confirm these results, cGAS protein expression was determined by Western blot in FLS from eight RA and OA patients. We found that the cGAS expression was significantly elevated in RA FLS as compared with the OA FLS (Figure 1(b)).

### 3.2. TNF*α* Stimulation Induced the cGAS Expression in RA FLS

**Figure 2:**
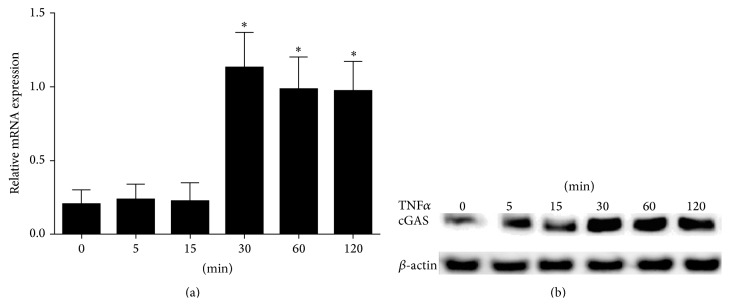
The expression of cGAS gene in rheumatoid arthritis (RA) fibroblast-like synoviocytes (FLS) was inhibited by tumor necrosis factor *α* (TNF*α*) inflammatory stimulation. (a) cGAS mRNA was upregulated by TNF*α* stimulation in a time-dependent manner. RA FLS (2 × 10^5^) were cultured in 6-well plates and then treated with TNF*α* (100 ng/mL) for different times (0, 15, 30, 60, and 120 min). (b) The level of cGAS protein was determined by Western blot. Values are the mean and SEM (standard error of the mean). Results are representative of 3 independent experiments. ^*∗*^ *P* < 0.01 versus 0 min.

TNF*α* is involved RA pathogenesis and TNF*α* inhibitors are now approved for the treatment of RA. In this study, we used the TNF*α* as the stimulus to investigate the cGAS expression in RA FLS. We showed that the expression level of cGAS mRNA was increased in a time-dependent manner in response to the stimulation with TNF*α* (100 ng/mL) by real-time quantitative PCR (Figure 2(a)). At 30 min of TNF*α* stimulation in our observations, cGAS mRNA expression was the highest (*P* < 0.01 as compared to the control, time 0). These observations were further validated at protein level by Western blot analysis (Figure 2(b)), suggesting that the expression of cGAS is augmented in RA FLS under proinflammatory stimulation by TNF*α*, implicating cGAS as a promoter of RA pathogenesis and progression. To test this assumption, we next explored the immunoregulatory role and mechanisms of cGAS overexpression, using recombinant lentivirus cGAS, in promoting the inflammatory responses of RA FLS.

### 3.3. Effect of cGAS on RA FLS Proliferation

**Figure 3:**
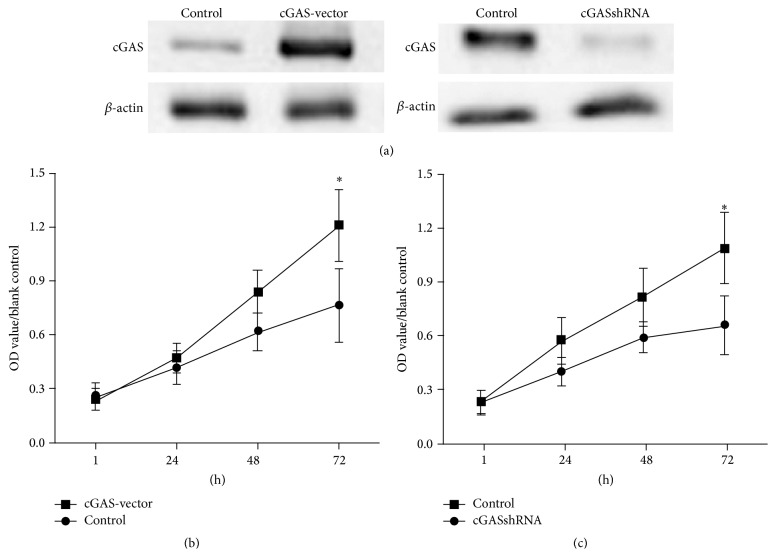
Effects of cGAS on RA FLS proliferation. (a) The transfection of cGAS expression vector enhanced cGAS protein levels, while the transfection of cGAS shRNA markedly reduced the protein levels of cGAS in the presence of TNF*α* stimulation for 30 min. (b) and (c) MTS assay showed that cGAS overexpression dramatically increased the proliferation of RA FLS, whereas cGAS knockdown reduced the proliferation of FLS. ^*∗*^ *P* < 0.01.

To evaluate the pathological significance of cGAS accumulation for RA progression, we modulated cGAS expression levels in RA-FLS by the transfection of the cells with cGAS expression plasmids or shRNA. As expected, the transfection of cGAS expression vector enhanced cGAS protein levels, while the transfection of cGAS shRNA markedly reduced the protein levels of cGAS in the presence of TNF*α* stimulation for 30 min (Figure 3(a)). MTS assay showed that cGAS overexpression dramatically increased the proliferation of RA FLS, whereas cGAS knockdown reduced the proliferation of FLS (Figures 3(b) and 3(c)).

### 3.4. Effect of cGAS Overexpression on TNF*α*-Induced Inflammatory Responses in RA FLS

**Figure 4:**
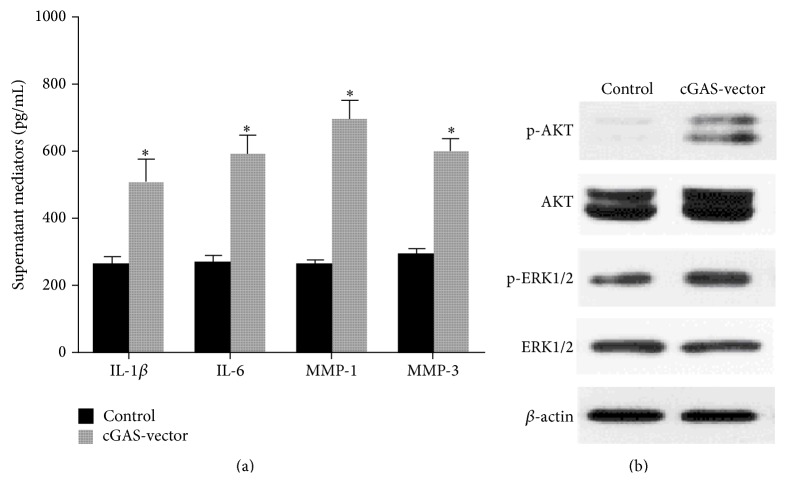
Effect of cGAS overexpression on TNF*α*-induced inflammatory responses in RA FLS. (a) FLS infected with cGAS-vector produced significantly higher amounts of IL-1*β*, IL-6, MMP-1, and MMP-3 than did FLS infected with control (^*∗*^ *P* < 0.01) when exposed to TNF*α* stimulation for 30 min. (b) Overexpression of cGAS significantly increased TNF*α*-mediated ERK1/2 and AKT phosphorylation by Western blotting.

We investigated the possible effects of cGAS on TNF*α*-induced production of key proinflammatory mediators, including cytokines IL-1*β*, IL-6, MMP-1, and MMP-3. The protein levels of above mediators in the supernatants of RA FLS grown in the absence or presence of TNF*α* were assessed by ELISA. The results showed that FLS infected with cGAS-vector produced significantly higher amounts of IL-1*β*, IL-6, MMP-1, and MMP-3 than did FLS infected with control (Figure 4(a), *P* < 0.01) when exposed to TNF*α* stimulation for 30 min. Activation of AKT or ERK pathway has been known to be associated with the inflammatory responses of FLS and RA. To further investigate the mechanisms by which cGAS promotes the inflammatory responses in RA FLS, including proinflammatory mediator production as described above, we detected the effect of cGAS on AKT and ERK pathways. We found that overexpression of cGAS significantly increased TNF*α*-mediated ERK1/2 and AKT phosphorylation by Western blotting (Figure 4(b)).

### 3.5. Effect of cGAS Knockdown on TNF*α*-Induced Inflammatory Responses in RA FLS

**Figure 5:**
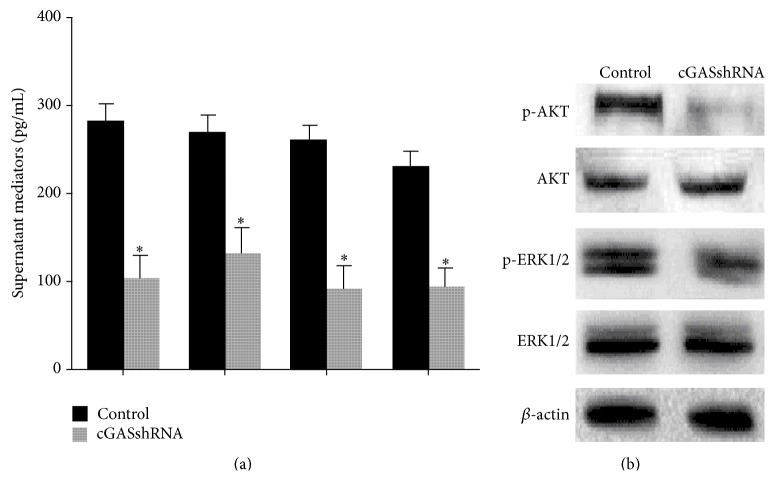
Effect of cGAS silencing on TNF*α*-induced inflammatory responses in RA FLS. (a) FLS infected with cGAS shRNA produced significantly lower amounts of IL-1*β*, IL-6, MMP-1, and MMP-3 than did FLS infected with control (^*∗*^ *P* < 0.01) when exposed to TNF*α* stimulation for 30 min. (b) Knockdown of cGAS significantly reduced TNF*α*-mediated ERK1/2 and AKT phosphorylation by Western blotting in the presence of TNF*α* stimulation for 30 min.

In addition, we investigated the possible effects of cGAS knockdown on TNF*α*-induced production of key proinflammatory mediators and AKT and ERK pathways. The results showed that FLS infected with cGAS shRNA produced significantly lower amounts of IL-1*β*, IL-6, MMP-1, and MMP-3 than did FLS infected with control (Figure 5(a), *P* < 0.01) when exposed to TNF*α* stimulation for 30 min. Activation of AKT or ERK pathway was also significantly diminished by cGAS shRNA by Western blotting in RA FLS in the presence of TNF*α* stimulation for 30 min (Figure 5(b)).

## 4. Discussion

TNF*α* is involved at each stage of RA pathogenesis, namely, by augmenting autoimmunity, sustaining long-term inflammatory synovitis, and promoting joint damage [15]. Five anti-TNF drugs, infliximab, adalimumab, certolizumab pegol, etanercept, and golimumab, are now approved for the treatment of RA in various countries [15]. In the present study, we employed the TNF*α*-induced RA FLS to simulate the in vitro model of RA. We found that cGAS was overexpressed in RA-FLS compared with OA FLS. TNF*α* stimulation induced cGAS expression in RA FLS. Overexpression of cGAS promoted the proliferation and knockdown of cGAS inhibited the proliferation of RA FLS. cGAS overexpression enhanced the production of proinflammatory cytokines and matrix metalloproteinases (MMPs) in TNF*α*-stimulated FLS. In contrast, cGAS silencing inhibited production of proinflammatory cytokines and matrix metalloproteinases (MMPs) in TNF*α*-stimulated FLS. These results suggest that cGAS activates the inflammatory response of RA FLS, and the development of strategies targeting cGAS may have therapeutic potential for human RA.

Accumulating data have implicated the activation of both AKT and ERK pathways in the pathogenesis of RA. AKT activation is an important pathophysiologic change associated with the proliferating synovium in RA. FLS from patients with RA express higher levels of phosphorylated AKT than those from patients with osteoarthritis [16]. PI3K/AKT signal pathway in RA FLS can be activated by proinflammatory cytokines such as TNF*α*, and AKT activation plays a crucial role in stimulating FLS proliferation and production of inflammatory cytokines (which perpetuate inflammation) and MMPs (which contribute to cartilage destruction) [17]. Correspondingly, downregulation of AKT activation results in the antiproliferative and anti-inflammatory effects in RA FLS and rat arthritis [18]. Other studies from experimental animal models of RA also demonstrate that antagonizing PI3K/AKT signaling cascades can promote joint inflammation, suggesting that AKT pathway may be a potential therapeutic target for RA [3].

ERK signal pathway is also involved in the activation of RA FLS and the destruction of bone in arthritic joints [19]. ERK is constitutively expressed in RA synovium and rheumatoid FLS, and the expression of the activated phosphorylated forms is much higher in RA synovium than in that of OA [20]. ERK signaling cascades are well known to play an important role in RA. Inhibition of Ras/ERK signal has been provided as a novel approach for RA treatment, by targeting FLS activation and bone destruction [19]. The Ras/ERK pathway inhibitor can block the production of inflammatory cytokines and MMPs by RA FLS and the invasiveness of FLS by antagonizing the activation of ERK [21]. Our data demonstrate that targeting cGAS inhibited ERK and AKT pathways, suggesting the potential role of cGAS in the pathogenesis of RA.

The novel finding of our study is that cGAS promotes the inflammation of RA. The potential role of cGAS in inflammatory and autoimmune disorders has recently been uncovered. Taken together, our results demonstrate a critical role of the cGAS/STING-mediated cytosolic DNA-sensing pathway for type I IFN induction in dendritic cells [20]. Antimalarial drugs inhibit IFN-*β* production through blockade of cGAS-DNA interaction [21]. Trex1 (-/-) mice lacking cGAS are completely protected from lethality, exhibit dramatically reduced tissue inflammation, and fail to develop autoantibodies, implicating cGAS as a key driver of autoimmune disease and suggesting that cGAS inhibitors may be useful therapeutics for Aicardi-Goutières syndrome and related autoimmune diseases [9]. In this study, we first determined the native expression of cGAS gene in inflammatory FLS from RA patients. In vitro, under inflammatory stimulation such as with TNF*α*, arthritic FLS exhibit inflammatory responses, and our studies showed that in this process cGAS expression was elevated with stimulation time both at mRNA and protein levels. Overall, our study highlights that cGAS is a proinflammatory factor by regulating ERK and AKT pathways, and this regulatory effect of cGAS is firstly reported in RA FLS.

In summary, we have demonstrated in this study that cGAS overexpression potently contributes to the inflammatory responses in RA FLS. The mechanisms of cGAS-mediated proinflammatory responses may lie in its modulation of both ERK and AKT pathways. The present study suggests that overexpression of cGAS or enhancement of cGAS activity in FLS may contribute a lot to the development of RA. The immunoregulatory strategy targeting cGAS might have therapeutic potential in the treatment of human RA.
